# Encouraging Family Forest Owners to Create Early Successional Wildlife Habitat in Southern New England

**DOI:** 10.1371/journal.pone.0089972

**Published:** 2014-02-26

**Authors:** Bill Buffum, Christopher Modisette, Scott R. McWilliams

**Affiliations:** 1 Department of Natural Resources Management, University of Rhode Island, Kingston, Rhode Island, United States of America; 2 Ecological Science, United Stated Department of Agriculture, Natural Resources Conservation Service, Warwick, Rhode Island, United States of America; 3 Department of Natural Resources Management, University of Rhode Island, Kingston, Rhode Island, United States of America; Lakehead University, Canada

## Abstract

Encouraging family forest owners to create early successional habitat is a high priority for wildlife conservation agencies in the northeastern USA, where most forest land is privately owned. Many studies have linked regional declines in wildlife populations to the loss of early successional habitat. The government provides financial incentives to create early successional habitat, but the number of family forest owners who actively manage their forests remains low. Several studies have analyzed participation of family forest owners in federal forestry programs, but no study to date has focused specifically on creation of wildlife habitat. The objective of our study was to analyze the experience of a group of wildlife-oriented family forest owners who were trained to create early successional habitat. This type of family forest owners represents a small portion of the total population of family forest owners, but we believe they can play an important role in creating wildlife habitat, so it is important to understand how outreach programs can best reach them. The respondents shared some characteristics but differed in terms of forest holdings, forestry experience and interest in earning forestry income. Despite their strong interest in wildlife, awareness about the importance of early successional habitat was low. Financial support from the federal government appeared to be important in motivating respondents to follow up after the training with activities on their own properties: 84% of respondents who had implemented activities received federal financial support and 47% would not have implemented the activities without financial assistance. In order to mobilize greater numbers of wildlife-oriented family forest owners to create early successional habitat we recommend focusing outreach efforts on increasing awareness about the importance of early successional habitat and the availability of technical and financial assistance.

## Introduction

Encouraging family forest owners (FFO) to manage their forests to create early successional habitat (ESH) is a high wildlife conservation priority in the northeastern United States [1]. Many studies have linked recent declines in early successional wildlife populations in the region to the loss of ESH [2]–[5]. FFOs, defined as families, individuals, estates, trusts, family partnerships, and other unincorporated groups of individuals owning at least 0.4 ha of forest land, control 55% of all forest land in Southern New England and a similar percentage in the 20 states in the northern United States [6], and thus could play an important role in creating ESH. The Natural Resources Conservation Service (NRCS) of the United States Department of Agriculture encourages FFOs to create ESH through financial and technical support programs such as the Wildlife Habitat Incentives Program and the Environmental Quality Incentives Program [7], [8]. However, NRCS funding to support forestry activities has not been fully utilized in recent years in states such as Rhode Island (RI); a missed opportunity for wildlife-oriented FFOs and wildlife conservation efforts in the state. Nationally only 6% of FFOs have participated in federal forestry financial assistance programs [9]. The number of landowners actively managing their forests in some states is decreasing [10]: with increasingly urban backgrounds and lifestyles, many forest owners see forestry as “irrelevant to their landowning objectives and immediate concerns” [11].

Several studies have analyzed participation of FFOs in NRCS forestry programs. None of these studies focused specifically on ESH, but some of the findings are relevant. For example, most participants in federal forestry programs were more concerned about “doing the right thing” than maximizing profits, but the financial incentives provided by the programs increased the number of acres treated [12]. However, landowners who were less financially dependent on their land appeared to be less interested in the financial incentives from the forestry programs [13], [14]. The likelihood of FFOs participating in forestry programs increased with the size of their forest holdings [9] and the number of years of ownership [15]. Many FFOs found the federal programs difficult to access and inflexible [16]. The likelihood of active forest management increased if the forest owners lived close to their forests [17].

The objective of our study was to analyze the experience of a group of FFOs who had a strong interest in wildlife and who had been trained to create ESH on their own properties. We conducted the study in RI, one of three states in the Southern New England region of the United States. RI is experiencing ongoing loss of ESH [18], and federal, state and private conservation groups are actively promoting forest management to create more habitat (AFA, 2010; Oehler, 2003; TNC, 2010; USFWS, 2008). RI is representative of Southern New England in terms of forest cover, forest land-use dynamics and forest ownership patterns: for example, FFOs own 57% of RI forests with average holdings of 2.4 ha, compared to 53% and 2.4 ha respectively in Massachusetts, and 50% and 3.6 ha in Connecticut [19].

In the current study we surveyed FFOs who participated in the RI Coverts Program, which has trained FFOs to create wildlife habitat on their own properties since 2008. These FFOs represent a small portion of the total population of FFOs, but we believe they can play an important role in creating wildlife habitat, and that it is important to understand how outreach programs can best reach them. Our study addressed three research questions. (a) What characteristics were shared by this group of FFOs (e.g. amount of forest holdings, prior experience with forestry, interest in earning income from their land)? (b) How important was technical and financial assistance in motivating these FFOs to create ESH? (c) What other factors made some of these FFOs more likely than others to follow up after the training with activities on their own land? To our knowledge, this was the first study that specifically examined the experience of FFOs in creating ESH. Even though our study had a small sample size and was limited to a subset of FFOs with a strong interest in wildlife conservation, our hope is that the results of the study can be used to strengthen forestry outreach programs in the region and encourage greater numbers of wildlife-oriented FFOs to create ESH on their own properties.

## Methods

### Ethics Statement

The University of Rhode Island (URI) Institutional Review Board (IRB) reviews research projects conducted at URI that involve human subjects to ensure that two broad standards are upheld: first, that subjects are not placed at undue risk; second, that they give uncoerced, informed consent to their participation. The application for the current study (Project Title: (239239-2) Study of forestry activities by private landowners in Rhode Island), was approved by the IRB on 6 September 2011. As per the approved IRB proposal, we requested the participants to formally consent to participating in the study. This was done by either checking a box on an on-line survey, or by consenting in writing if they decided to submit a paper version of the survey rather than the on-line version. We retained the records for all participants.

### Data Collection

We surveyed all FFOs who attended the three day RI Coverts Program from 2008 to 2012. A total of 79 persons representing 54 households attended from 2008 to 2012. We sent one survey per household, and did not include program participants who were not FFOs, such as representatives of conservation agencies. Before distributing the 54 surveys, we announced the study in a newsletter to FFOs distributed by NRCS. We then contacted the program participants by email or regular mail, using the contact information they provided when they registered for the program. We gave the respondents the option of filling out paper or online versions of the survey. The survey included 41 questions that were a mix of multiple-choice questions to generate quantitative data as well as open ended questions to generate qualitative data. The survey included three components: (a) general demographics and interest in forestry and wildlife, (b) experience with forestry activities before attending the program, and (c) experience with forestry activities after the training. In the summer of 2011 we surveyed all FFOs who participated in the program between 2008 and 2011. In the summer of 2012 we distributed a shorter version of the survey to the participants of the 2012 program - this version included the first two components of the original survey, but not the section on experience with activities after the training.

### Data Analysis

Our small sample size (N = 34) limited the options for statistical analysis, but we used SPSS v. 20 to calculate the Likelihood Ratio Statistic (*LX^2^*) when our data met the requirements for expected frequencies.

## Results

The total number of respondents was 34, of whom 19 had implemented activities after attending the program. The overall response rate was 63%. Ninety four per cent of the respondents filled out an on-line survey, and 6% filled out printed surveys. The quantitative results of the survey are presented in Tables 1 and 2, and the qualitative results from open ended questions are presented in the narrative.

**Table 1:** Attributes of Family Forest Owners (FFO) who attended RI Coverts Program (N = 34).

	Percent of FFOs
Area of forest ownership: 0.4–3.6 ha	13%
Area of forest ownership: 4–19 ha	38%
Area of forest ownership: 20–39 ha	19%
Area of forest ownership: 40+ ha	31%
Duration of forest ownership: <10 years	15%
Duration of forest ownership: 10–24 years	46%
Duration of forest ownership: 25–50 years	31%
Duration of forest ownership: >50 years	8%
Have some university education	100%
Interest in earning forestry income: Moderate/strong	65%
Interest in earning forestry income: Slight/none	35%
Interest in wildlife: Observe wildlife	94%
Interest in wildlife: Identify species of wild plants or animals	88%
Interest in wildlife: Hunt/fish on own property	47%
Participate in forest certification program	69%
Participate in forest easement program	31%
Before attending Coverts Program: Received forestry advice	71%
Before attending Coverts Program: Started to prepare management plan	62%
Before attending Coverts Program: Hired a forester	62%
Before attending Coverts Program: Implemented some forestry activity	74%
Before attending Coverts Program: Hired a logger	32%
Before attending Coverts Program: Logged without paid help	24%
Before attending Coverts Program: Sold timber or firewood	32%
Before attending Coverts Program: Harvested timber or firewood for personal use	50%
Before attending Coverts Program: Created openings for ESH	38%
Involvement in management of other land: Conservation organizations	38%
Involvement in management of other land: Land of friends and relatives	22%

**Table 2:** Attributes of Family Forest Owners (FFO) who implemented forestry activities on their properties after attending Coverts Program (N = 19).

	Percent of FFOs
After attending program: Started forest management plan	32%
After attending program: Hired a forester	42%
After attending program: Harvested timber or firewood for personal use	74%
After attending program: Created forest openings	79%
After attending program: Hired a logger	21%
After attending program: Sold timber or firewood	32%
After attending program: Logged without any paid help	42%
After attending program: Received NRCS financial support	84%
Would probably not have implemented activities without financial support	47%
Would probably not have implemented activities without attending the program	33%
Plan to request future NRCS financial support	79%
Feel that the implementation was harder than expected	20%
Feel that the implementation was easier than expected	20%
Feel that they need additional wildlife/forestry training	53%
Can describe positive impact of implemented activities on wildlife	68%

### Forest Ownership

Fifty percent of our respondents owned more than 20 ha of forest (Table 1). The respondents mentioned several advantages of having larger forest holdings when creating ESH: greater flexibility in site selection for forest management, fewer conflicts with neighbors who resented clearcuts near their property boundaries, and greater ability to engage loggers who preferred larger jobs. Thirteen percent of the respondents owned less than 4 ha of forest. The respondents who owned less than 20 ha were less likely than respondents with larger forest holdings to have a moderate or strong interest in earning income from their forests (*LX^2^*(1) = 7.197, n = 34, p<0.01) and to have sold timber or firewood before attending the Coverts Workshop (*LX^2^*(1) = 6.983, n = 34, p<0.01). However, there were no significant differences in their likelihood of having engaged in forest management activities before attending the Coverts Program (hiring a forester, preparing a management plan, or harvesting timber or firewood for their personal use) or having followed up after attending the Coverts Program with forest management activities on their own land (*LX^2^*(1) = 0.125, *LX^2^*(1) = 0.125, (*LX^2^*(1) = 0.118, *LX^2^* (1) = 0.000 respectively; p>0.5 in all cases).

### Networking with Other Forest Owners

Before attending the Coverts Program, 71% of the respondents had already received some forestry advice from friends or relatives and 62% had hired a professional forester. Sixty nine percent were involved in forest certification programs such as the Rhode Island Tree Farm Program, 31% were involved in forest easement programs, and 62% had started to prepare a forest management plan. In addition to managing their own forests, 60% were involved in planning forest management activities on land that did not belong to them, such as land owned by friends, relatives or conservation organizations.

### Knowledge about Early Successional Habitat

All of our respondents expressed a strong interest in wildlife, whether observing wildlife, hunting, or identifying species of wild plants and animals. Sixty eight percent of the respondents who had implemented activities since attending the program could describe some positive impact of their forest management activities, including increased abundance of birds, mammals, amphibians and insects. Awareness about the importance of ESH for wildlife was low: several respondents commented that before attending the program they did not realize that many wildlife species depend on ESH or that this habitat type was declining in New England. Several commented that they viewed clearcutting negatively before visiting other FFOs who had already created ESH. Thirty three percent of the respondents who implemented activities after attending the program said they probably would not have implemented the forestry activities if they had not attended the program.

### Forest Owner Follow-up after Attending Training

Eighty three percent of our respondents who had attended training at least six months before the survey had already followed up with forest management activities on their own land, and all of the other respondents were planning to implement activities. The respondents implemented a range of forest management activities after attending the Coverts Program (Table 2). The most common activity was creating forest openings to generate ESH, with opening sizes ranging from 0.2 ha to more than 6 ha per household. None of the personal attributes recorded in the study were significantly related to how quickly the participants initiated activities on their own land. However, 80% of respondents with management plans had implemented additional forest management activities after the training, whereas none without a forest management plan had implemented any activity since the training except for starting to prepare a management plan (which can take up to one year).

### Importance of NRCS Financial Support

Our respondents varied in terms of interest in earning income from their forests: 12% were not at all interested in earning forest income, while 24% were slightly interested and 65% were at least moderately interested. However, the financial and technical assistance offered by NRCS programs appeared to be an important motivating factor for our respondents. Eighty four per cent of our respondents who had implemented activities after attending the RI Coverts Program had received support from NRCS, and 47% said they would probably not have implemented the activities without the financial assistance. Several respondents mentioned that the process of obtaining NRCS support was complex and time consuming, and/or that they were not aware about these financial assistance programs before attending the Coverts Program.

## Discussion

Our findings demonstrate that this subset of FFOs shared some common attributes such as education level and interest in wildlife, but differed in terms of land holdings, prior experience with implementing forestry activities, and interest in earning income from their forests.

### Forest Ownership

The FFOs in our study tended to have large forest holdings: half owned more than 20 ha of forest, whereas the National Woodland Owner Survey (NWOS) conducted by the US Forest Service reported that only 3% of FFOs in Southern New England and 0% in RI owned more than 20 ha of forest [20]. (The NWOS included 33 respondents in RI, but the area-based sampling frame resulted in very high sampling errors for most attributes [21] so we compared our results to the NWOS results for Southern New England, which included 887 respondents). Our respondents mentioned several advantages of having larger forest holdings when creating ESH, such as greater flexibility in site selection, fewer conflicts with neighbors, and greater ability to engage foresters and loggers. Other studies have reported correlations between larger forest holdings and more active forest management [17], [20] and greater participation in forestry programs [15], [22]. However, half of our respondents had forest holdings under 20 ha, including 13% with holdings under 4 ha as is more typical for the region (90% of NWOS Southern New England respondents). We found that FFOs with forest holdings under 20 ha were less likely than other respondents to be interested in earning income from their forests, but not significantly less likely to have actively managed their forests before or after attending the Coverts Program. Recent forestry outreach experience in RI has confirmed that FFOs with small forest holdings are interested in forest management: a series of lectures on forest management supported by NRCS in 2013 attracted 65 FFOs, of whom 31% owned no more than 4 ha of forest and 55% owned no more than 8 ha (Sayles, K. 2013, unpublished data). These findings suggest an opportunity for outreach programs in Southern New England to target FFOs with small forest holdings. These FFOs can make a valuable contribution by creating small openings of 0.6 ha or more, which provide suitable habitat for many shrubland bird species [23], and can contribute to creating habitat for species requiring larger habitat patches, such as New England Cottontail, if their properties are close to existing patches of habitat. These FFOs are eligible to participate in NRCS forestry programs, which, unlike some state forestry programs, do not require that participants own at least 4 ha of forest.

### Knowledge about Early Successional Habitat

The strong interest in wildlife expressed by the respondents was not surprising since they had volunteered to participate in a three day training on wildlife issues. More unexpected was their limited understanding about the importance of ESH prior to the training. Several respondents commented that before attending the program they were unaware that many wildlife species depend on ESH and that this habitat type is declining in New England. This applied to several respondents who had already prepared forest management plans before attending the training, which suggests that the consulting forester did not stress the importance of creating ESH during the process of plan preparation. Several respondents commented that they felt negatively about forest clearcutting before attending the Coverts Program, an attitude that is common in other Southern New England states [10]. These findings highlight the importance of educating forest owners about ESH. If FFOs with a strong interest in wildlife, such as our respondents, are unaware of the importance of early successional habit, it seems likely that the awareness of most FFOs is even lower.

### Networking with Other Forest Owners

Our respondents appeared to be comfortable networking with other FFOs and receiving advice about forest management. Even before attending the Coverts program, most had received forestry advice from friends, relatives or professional foresters, and many had participated in forest certification programs. Other studies have reported that land owners who receive advice about their forests from professionals or friends are more likely to participate in forestry support programs [15], [22] and that participation in a forest certification program may allow landowners to become more comfortable with harvesting trees [10]. Several respondents commented that they learned the most during the Coverts Program from field trips to other FFOs who had already implemented forest management activities on their own properties. The benefits of peer- to-peer learning have been emphasized by several authors as an effective approach to motivate forest owners to manage their forests [17], [24]. Our findings suggest that outreach programs should take advantage of existing networks of wildlife-oriented FFOs such as conservation organizations and land trusts to identify FFOs who can potentially be motivated to create ESH on their own properties, and that the outreach programs should promote peer-to-peer learning by expanding the number of forestry events located on land of FFOs who have already implemented forest management activities.

### Follow Up after the Training

The main factor affecting how quickly the respondents implemented forest management activities after attending the training appeared to be whether or not the respondent had prepared a forest management plan, a process which can take up to a year. Eighty per cent of the respondents with management plans had implemented additional forest management activities after the training, whereas none without a forest management plan had implemented any activity after the training except for preparing a management plan. As such, we endorse the current NRCS program of providing technical and financial support to encourage FFOs to prepare forest management plans, an important first step in creating wildlife habitat.

### Importance of NRCS Financial Support

Our respondents varied in terms of interest in earning income from their forests, but the financial and technical assistance offered by NRCS programs appeared to be an important motivating factor for them. Most respondents who had implemented activities after attending the RI Coverts Program received financial assistance from NRCS and would probably not have implemented the activities without this assistance. We did not see any indications that landowners who are less interested in earning income from their forests were less interested in the financial incentives, as was reported in North Carolina [13]. Several respondents commented that they were disappointed with the low payment offered by loggers when creating ESH, which is not surprising as the annual median stumpage value of hardwood firewood in Rhode Island ranged from $5 to $10 per cord during the study period [25]. Our findings agree with Daniels et al. [12], who reported that profit was not the primary objective of many forest owners, but that the financial incentives increased the area of forest the owners were willing to manage.

However, several respondents commented that they did not know about the NRCS financial assistance programs before attending the Coverts Training. Other respondents mentioned that the process of obtaining NRCS support was complex and time consuming, which is a common complaint of landowners about federal financial assistance programs [16]. Clearly, these important technical and financial assistance programs need to be promoted more widely, and training materials need to be developed that describe the application process in a way that is easier for FFOs to understand.

## Conclusions

Our study examined the characteristics of FFOs who have a strong interest in wildlife. We found that this subset of FFOs share some characteristics such as education level, but differ in terms of land holdings and interest in earning income from their forests. Most had prior experience with implementing forestry activities, but 26% were totally unengaged in forest management before attending the program. A key finding of our study was that despite their strong interest in wildlife, many respondents had not been aware before attending the Coverts Program of either the importance of ESH or the availability of financial support currently available from NRCS. However, these FFOs were willing to implement forest management activities on their own land once they were provided with adequate training and financial incentives. These FFOs represent a small portion of the total population of FFOs, but we believe they can play an important role in creating wildlife habitat, and we recommend the expansion of outreach programs such as the Coverts Program. The sample size of our study was smaller than we would have liked, and we recommend conducting a comparable study with a larger sample size to confirm our findings. Meanwhile we offer these preliminary recommendations for an outreach strategy to motivate greater numbers of wildlife-oriented FFOs to engage in forest management: (a) use existing networks of wildlife-oriented FFOs such as conservation organizations and land trusts to identify FFOs who are not yet engaged in forest management but who can potentially be motivated to create ESH on their own properties; (b) focus the content of outreach efforts on the importance of creating ESH and the availability of NRCS technical and financial assistance; (c) encourage peer-to-peer learning by providing more opportunities for FFOs to visit FFOs who have already created ESH on their own land; (d) develop simpler descriptions of NRCS forestry programs and application procedures to make them more easily understood by FFOs; (e) encourage FFOs with smaller forest holdings (4 ha or less) to manage their forests: these FFOs can make a valuable contribution by creating small patches of ESH which provide critical habitat for many species of shrubland birds [23]; and (f) encourage consultant foresters to educate FFOs about the need for ESH by providing refresher trainings for consultant foresters on wildlife issues such as the minimum opening size required by different wildlife species.
